# Suspicious Case of a Body packer “Mule” in a Low Resource Country: A Case Report

**DOI:** 10.31729/jnma.6618

**Published:** 2021-06-30

**Authors:** Bibek Rajbhandari, Olita Shilpakar, Subash Thapa, Sumi Singh

**Affiliations:** 1 Department of General Practice and Emergency Medicine, Nepal Police Hospital, Kathmandu, Nepal; 2 Department of General Practice and Emergency Medicine, NAMS Bir Hospital, Kathmandu, Nepal; 3 Department of Radiology, Nepal Police Hospital, Kathmandu, Nepal; 4 Nepal Medical College, Kathmandu, Nepal

**Keywords:** *complications*, *custody*, *illicit drugs*, *lethal*

## Abstract

Body packing is the process of smuggling illicit drugs in the form of packages concealed within the gastrointestinal tract via ingestion or inserting into body orifices. These individuals are described as "body packers", "stuffers", "mules" or "swallowers" and resort to carrying drugs like heroin, cocaine and cannabis. They present to the hospital following the development of complications or brought dead due to the rupture of packets or directly from detention for further investigations. This case illustrates a suspected case detained from the airport who was found to be carrying 93 pellets of an illicit drug, heroin, weighing 900 grams, one of the highest quantity carried by any body packer in the country till date. This case further sheds light on the fact that a meticulous history, detailed clinical examination and radiographic investigations like abdominal radiograph and imaging are the keys to diagnose body packers in a resource limited setting.

## INTRODUCTION

Drug trafficking by internal body concealment was first reported in literature in 1975 and is a growing issue internationally.^1^ Body packers stuff their bodies with illicit drugs that most commonly include heroin, cocaine, hashish and other derivatives of cannabis via oral intake or by hiding inside anatomical orifices like rectum, vagina, intestine, ear, etc. Obstruction, perforation or even death are the common complications encountered in such mules in case of rupture of the packets since the dose accommodated in them could exceed the toxic dose for humans by multiple times.^2^ We describe the case of an asymptomatic body packer who was diagnosed by a detailed history, physical examination and radiological findings and was effectively managed with conservative measures.

## CASE REPORT

A 49-year-old male was brought to the Emergency Department (ED) by the airport police officials with a high suspicion of carrying illegal drugs. He was detained in the airport following some relevant piece of information regarding his destination. This was further confirmed by the suspicious behavior of the person in the form of restlessness, not eating or drinking anything or going to the toilet and furthermore, holding his abdomen throughout the flight. On arrival in the ED, he denied history of ingestion of any illicit drugs, however complained of nausea and abdominal fullness. There was no vomiting, chest pain, cough, shortness of breath or seizures. He was under medication for hypertension since the past 10 years. The patient reluctantly consented for a general physical examination. He was well oriented to time place and person but became increasingly anxious during the examination and started sweating excessively. His vitals showed an increase in blood pressure of 170/100mm Hg, a tachycardic pulse rate of 114 beats per minute, respiratory rate 20 breaths per minute, temperature 98 degrees Fahrenheit and an oxygen saturation of 95% in room air. A distended but soft and non-tender abdomen was revealed during his gastrointestinal examination. A grossly intact neurology with reactive pupils bilaterally and unremarkable findings in the respiratory and cardiovascular systems were evident.

**Figure 1:**
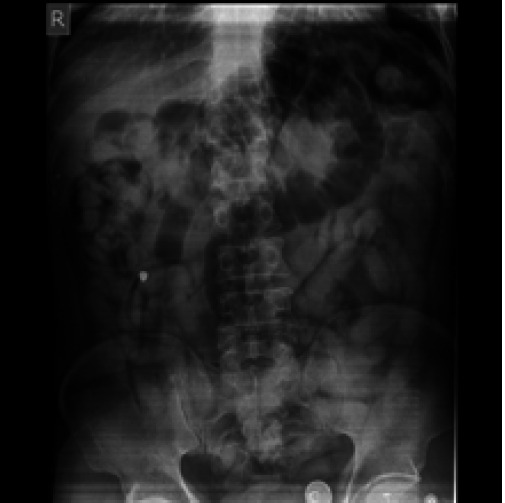
Abdominal radiograph showing multiple well circumscribed foreign bodies.

His abdominal radiograph revealed striking features of multiple well circumscribed similar sized elongated radiopaque shadows with a thin peripheral rim of lucency (Figure 1).

**Figure 2:**
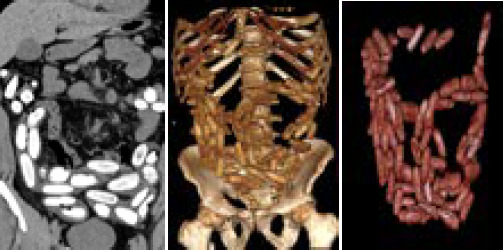
A. CT abdomen-coronal image, B. 3D volume rendering image, C. 3D volume rendering image with bone subtraction.

A non-contrast CT abdomen confirmed images of multiple pellet sized hyperdense foreign bodies throughout the bowel without any signs of obstruction or perforation. These individually measured approximately 4.5 x 1.5cm in size with HU of approximately +222, consistent with cocaine pellets (Figure 2A, 2B, 2C).

A chest radiograph was normal and a 12 lead electrocardiogram revealed sinus tachycardia of 110 beats per minute. His hemogram, renal and liver functions random blood sugar, coagulation profile, urinalysis and an arterial blood gas analysis were all within normal ranges.

**Figure 3:**
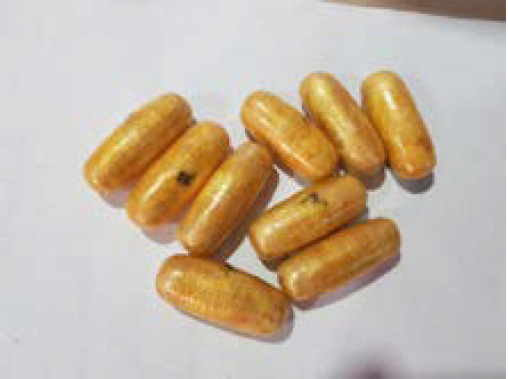
The retrieved packets of heroin.

Upon questioning him again after having explained the findings revealed by the abdominal radiograph and the CT scan and the possibility of fatal complications of the illicit drugs to his body, the person finally admitted having ingested 93 packets of the drug. He was kept under close observation with constant monitoring of his vitals. The patient was given 15 ml of lactulose twice over a span of 12 hours following which he expelled a total of 93 pellets in five batches 48 hours after ingestion of the drug packets (Figure 3).

Each pellet measured approximately 3.5cm x 1.5cm using a vernier calliper and accomodated a total weight of 900 grams of the illicit drug. On examining the retrieved pellets thoroughly, the reports of the investigation bureau revealed a white powdery substance identified as heroin covered in multiple tightly packed layers of latex like material.

His vitals were maintained throughout his stay in the hospital. He was taken into custody for further trials the next morning. At the time of discharge, he was asymptomatic with a blood pressure 130/90mm Hg, pulse 84/min, respiratory rate 18/min and saturation 95% in room air. He was doing well during a follow up appointment in the outpatient department after three days.

## DISCUSSION

Body packing is one of the common means of transporting illegal drugs across high security ports worldwide by drug traffickers since many decades. The emergence of body packers date back to the early seventies where predominantly young men were used for this purpose, however, there are evidences of adolescent males and pregnant women being used for the same.^2,3^

Heroin, cocaine and cannabis are the most commonly used drugs, earlier with precarious packaging to sophisticated automated ones these days which use waterproof multilayered latex sheath of balloons or condoms. The size and weight of the packets depend upon the type of the illicit drug, the size of the body packer and the materials and skills of packaging.^3^ Loosely packed drugs are highly prone to rupture and lead to life threatening consequences progressing to death. Olumbe, et al. reports a case of a female body packer who died due to the effect of ruptured heroin packets concealed in her gastrointestinal tract.^4^

A detailed history regarding the symptoms, namely pain abdomen, distension, nausea vomiting, constipation should be taken. Information regarding the number of packets ingested or inserted in any orifices and the type of the drug should be inquired though in most of the cases honest confession by the detainee is rare. A thorough physical examination should include the vitals, mental status of the patient, bowel sounds and pupil size in view of any toxic effects induced by the drug. Altered mental staus, miosis, decreased bowel sounds followed by respiratory depression are some of the important signs of heroin toxicity.^2^ Immediate measures need to be taken to combat these side effects. Our case did not have any signs of heroin toxicity, but a case report by Vahabzadeh, et al. states a heroin body packer with signs of overdose managed successfully with progressively increasing doses of naloxone.^5^

A plain abdominal radiograph is one of the most widely available, cost effective and non-invasive methods for screening body packers with a sensitivity of almost 85 to 90%. The drug packets may appear on a plain abdominal radiograph as multiple, radiopaque similar sized shadows; packets arranged in parallel with the bowel lumen known as "parallelism"- thin rim of air trapped between the wrapping layers, the "double condom sign"- multiple cylindrical shaped packets throughout the stomach and bowel, the "tictac sign" and air trapped at the tapered end of the packets, the "rosette sign". One or more of these signs confirm the diagnosis of a body packer by abdominal radiograph.^6^ CT scan is more sensitive than abdominal x-ray to determine a body packer since it identifies the different types of drugs on the basis of differences in the Hounsfield units with heroin having a value of i520 and cocaine with i219.^3,6,7^ However, a case study reports pitfall due to a foreign body of similar density mimicking the image of the drug packet located in the stomach.^8^ Our case also suggested the drug to be cocaine initially as per the density of the image, later revealed as heroin following numerous tests by the investigation bureau. Urine toxicology has also been used in a few cases for initial evaluation, but not recommended for routine use due to poor of sensitivity of this test.^3^

Body packers have been found to present own self to medical facilities after complaining of symptoms of mechanical obstruction and have been diagnosed with acute bowel obstruction or perforation. Surgical intervention with exploratory laparotomy in patients with signs and symptoms of obstruction and drug overdose has been beneficial if performed in time.^9^ Asymptomatic body packers rarely present to the hospital, except in detained cases brought by the police officials for a thorough assessment as a part of the legal procedure. An adequate observation time is advised even if the body packer does not show signs of complications and close monitoring is required to detect any signs of rupture of the packets to enable early treatment. A soft light diet and laxatives have been used in many cases to assist spontaneous expulsion of the packets, however some oil-based lubricants may have an adverse effect on the latex condoms resulting in rupture due to their property to decrease the tensile strength of these coverings. In the asymptomatic ones or in patients with mild symptoms like abdominal fullness or nausea, medical management of the symptoms is recommended.^10^

This case report illustrates an interesting case of body stuffing with heroin. Detailed histories, clinical examination aided by radiological investigations are the fundamentals in diagnosing a body packer. Since this method of illicit drug smuggling is growingly popular, it demands a high level of awareness and expertise among the treating clinicians for a favorable outcome.
